# PSMA ligands for diagnosis and therapy of prostate cancer

**DOI:** 10.1186/1470-7330-14-S1-O10

**Published:** 2014-10-09

**Authors:** Uwe Haberkorn

**Affiliations:** 1 Dept. of Nuclear Medicine, University Hospital Heidelberg and DKFZ Heidelberg, Germany

## 

Since the prostate-specific membrane antigen (PSMA) is frequently over-expressed in prostate cancer (PCa) several PSMA-targeting molecules are under development to detect and treat metastatic castration resistant prostate cancer (mCRPC).

We investigated 319 patients who received a ^68^Ga-PSMA^HBED^-PET/CT. In 82.8% of the patients at least one lesion indicative for PCa was detected. Tumor detection was positively associated with PSA level and androgen deprivation therapy (ADT). Mean SUVmax of analyzed tumor lesions was 13.3 ± 14.6. Amongst lesions investigated by histology, 30 were false-negative in ^68^Ga-PSMA^HBED^-PET/CT (one local relapse in one patient and 29 lymph nodes in another patient), all other lesions (n=416) were diagnosed true-positive or -negative. Fifty of 116 patients available for follow-up received local therapy after ^68^Ga-PSMA^HBED^-PET/CT.

In thirty-seven patients with biochemical relapse of PC a comparison was done between ^18^F-fluoromethylcholine- and ^68^Ga-PSMA-PET/CT within a time window of 30 days. Radiotracer uptake that was visually considered as PC lesion was subsequently semi-quantitatively analyzed by measuring the SUV_max_ values of the scans acquired 1 hour p.i. of ^68^Ga-PSMA complex solution (59 - 263 MBq, median of 132 MBq) and ^18^F-fluoromethylcholine (114 - 374 MBq, median of 237 MBq) respectively. In addition tumor to background ratios were calculated. 78 PC-suspicious lesions were detected in 32 patients using ^68^Ga-PSMA-PET/CT and 56 lesions were detected in 26 patients using Choline-PET/CT. The higher detection rate in ^68^Ga-PSMA-PET/CT concerning PC-suspicious lesions was significant (p=0.04). In 5 patients no lesion was found. All lesions detected by ^18^F-fluoromethylcholine-PET/CT were also seen by ^68^Ga-PSMA-PET/CT. In ^68^Ga-PSMA-PET/CT SUV_max_ was clearly (>10%) higher in 62 of 78 lesions (79.1%) and tumor-to-background ratio was clearly (>10%) higher in 73 of 78 lesions (93.6%) when compared to ^18^F-fluoromethylcholine-PET/CT.

Since the ligand bound to PSMA is internalized, the target may also be used for endoradiotherapy. We investigated the tissue kinetics of a small molecule inhibitor of PSMA ((*S*)-2-(3-((*S*)-1-carboxy-5-(3-(4-[^124^I]iodophenyl)ureido)pentyl)ureido)pentanedioicacid; MIP-1095) using PET/CT to estimate radiation dosimetry for the potential therapeutic use of ^131^I-MIP-1095 in men with mCRPC. We also report preliminary safety and efficacy of the first 28 consecutive patients treated under a compassionate use protocol with a single cycle of ^131^I-MIP-1095.

I-124-MIP-1095 PET/CT images showed excellent tumor uptake and moderate uptake in liver, proximal intestine and within a few hours post-injection also in the kidneys. High uptake values were observed only in salivary and lacrimal glands. Dosimetry estimates for I-131-MIP-1095 revealed that the highest absorbed doses were delivered to the salivary glands (3.8 mSv/MBq, liver (1.7 mSv/MBq) and kidneys (1.4 mSv/MBq). The absorbed dose calculated for the red marrow was 0.37 mSv/MBq. PSA values decreased by >50% in 60.7% of the men treated. 84.6% of men with bone pain showed complete or moderate reduction in pain. Hematological toxicities were mild. 25% of men treated had a transient slight to moderate dry mouth. No adverse effects on renal function were observed.

Based on the biodistribution and dose calculations of the PSMA-targeted small molecule ^124^I-MIP-1095 therapy with the authentic analog ^131^I-MIP-1095 enables a targeted tumor therapy with unprecedented doses delivered to the tumor lesions. Involved lymph node and bone metastases were exposed to estimated absorbed doses upwards of 300 Gy.

